# Complexes of *myo*-inositol-hexakisphosphate (InsP6) with zinc or lanthanum to enhance excretion of radioactive strontium from the body

**DOI:** 10.1371/journal.pone.0195067

**Published:** 2018-04-03

**Authors:** Kazuma Ogawa, Miho Aoki, Sumi Kadono, Akira Odani

**Affiliations:** 1 Institute for Frontier Science Initiative, Kanazawa University, Kanazawa, Japan; 2 Graduate School of Medical Sciences, Kanazawa University, Kanazawa, Japan; Pennsylvania State University, UNITED STATES

## Abstract

^90^Sr, which was released into the atmosphere and the ocean following the Chernobyl and Fukushima Daiichi nuclear power plant disasters, is an important nuclear fission element. Compounds that inhibit the absorption of ^90^Sr into the bloodstream and enhance its elimination can be beneficial in decreasing the absorbed radiation dose in people exposed to ^90^Sr. Recently, we prepared complexes of *myo*-inositol-hexakisphosphate (InsP6) with zinc or lanthanum as decorporation agents. These complexes, called Zn-InsP6 and La-InsP6 respectively, are insoluble in water and can potentially chelate additional metal cations. Hypothesizing that these complexes can assist the excretion of ^90^Sr from the body, we evaluated them using ^85^Sr instead of ^90^Sr. In *in vitro* binding experiments, Zn-InsP6 showed higher strontium adsorption capacity than La-InsP6. We then performed *in vivo* biodistribution experiments of Zn-InsP6 in mice after oral administration of ^85^SrCl_2_. Mice treated with Zn-InsP6 showed significantly lower bone accumulation of radioactivity than mice in a non-treatment control group. Zn-InsP6 adsorbed radiostrontium in the gastrointestinal tract, inhibited this ion’s absorption into the bloodstream, and enhanced its excretion in the feces. Therefore, Zn-InsP6 appears to be a promising ^90^Sr “decorporation” agent.

## Introduction

Strontium, an alkaline earth metal, acts as a calcium mimic and highly accumulates in bone [1]. Different types of radiostrontium exist. ^89^Sr (t_1/2_ = 50.5 d) as ^89^SrCl_2_ has been administered as a pain palliation for patients with bone metastases of cancer [2]. Following the intravenous injection of ^89^SrCl_2_ solution, ^89^Sr^2+^ is incorporated into collagen mineralization during new bone formation, so accumulates at high levels at the sites of bone metastases. Consequently, the intense pain associated with bone cancer is diminished by the radiation emitted by ^89^Sr. On the other hand, ^90^Sr (t_1/2_ = 29.1 y) is one of the most important nuclear fission elements. After the nuclear power plant disasters of Chernobyl and Fukushima Daiichi, ^89^Sr and ^90^Sr were released into the atmosphere and the ocean [3–6]. ^90^Sr is more harmful to humans than ^89^Sr, because ^90^Sr has a much longer half-life, and its daughter radionuclide, ^90^Y (t_1/2_ = 64.1 h), emits high-energy beta particles. It has been reported that internal exposure to ^90^Sr could be associated with the development of leukemia and osteosarcoma [7,8]. Therefore, compounds inhibiting the absorption of radiostrontium from the gastrointestinal tract into the bloodstream and enhancing its elimination after intake can decrease the absorbed radiation dose of people exposed to radiostrontium. Indeed, basic research has shown that alginate can promote the excretion of ^90^Sr [9,10].

**Fig 1:**
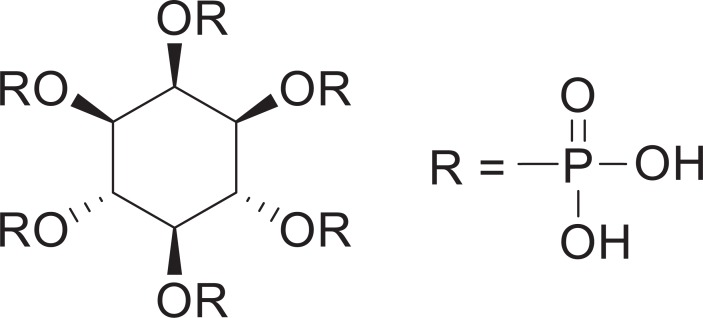
The structure of *myo*-inositol-hexakisphosphate (InsP6).

*Myo*-inositol-hexakisphosphate (phytic acid: InsP6, Fig 1) is a natural compound that abounds in plants, especially in whole grains, cereals, legumes, seeds, and nuts [11]. Due to its structure, InsP6 exhibits high chelation potential with many kinds of metal cations [12,13]. Recently, we prepared complexes of InsP6 with zinc or lanthanum ions (Zn-InsP6 and La-InsP6, respectively) and evaluated them as radiocesium “decorporation” agents, because both complexes are insoluble in water and are spacious enough to potentially accommodate additional metal cations coordinated by chelation. In fact, InsP6 is soluble in water, but chelation with zinc or lanthanum ions decreases the solubility in water, and thereby reduces the absorption in the intestinal tract.

In this study, we hypothesized that Zn-InsP6 and La-InsP6 can work as ^90^Sr decorporation agents and evaluated their potential *in vitro* and *in vivo*. Experiments using normal mice were performed to evaluate the influence of Zn-InsP6 on the biodistribution of radiostrontium. In these experiments, we replaced ^90^Sr with ^85^Sr (T_1/2_ = 64.8 d) because ^85^Sr emits gamma rays, which are easy to measure.

## Materials and methods

### Materials

^85^SrCl_2_ (> 111 GBq/g) was purchased from PerkinElmer (Waltham, MA, USA). InsP6 was purchased from Sigma-Aldrich (St. Louis, MO, USA) as phytic acid sodium salt hydrate, InsP6•6Na^+^•6H_2_O. Other reagents were of reagent grade and used as received. All sections of this report adhere to the ARRIVE Guidelines for reporting animal research [14]. A completed ARRIVE guidelines checklist is included in supporting information (S1 File).

### *In vitro* adsorption of ^85^Sr to La-InsP6 and Zn-InsP6

La-InsP6 and Zn-InsP6 were prepared by the method previously reported [15]. La-InsP6 (La: InsP6 = 2: 1) and Zn-InsP6 (Zn: InsP6 = 2: 1) were used for *in vitro* and *in vivo* experiments. La-InsP6 and Zn-InsP6 samples (10 mg/mL) containing various concentrations of Sr and 10 kBq/mL of ^85^Sr, in 20 mM HEPES buffer (pH 7.4) containing 10 mM Na^+^, were prepared. After shaking at 1,000 rpm and 37°C for 1 h, the samples were centrifuged at 10,000*g* and room temperature for 10 min. The radioactivity of the supernatant was measured using an auto-well gamma counter (ARC-380; Hitachi healthcare, Ltd., Tokyo, Japan), and the counts were corrected for background radiation. Control experiments were performed using the same procedure but without M-InsP6 (M = Zn or La). The binding ratios were determined as follows:

BindingratiotoM‑InsP6(%)=(1−[radioactivityofsupernatantofeachsample]/[radioactivityofsupernatantintherespectivecontrol])×100

### Effects of cations (Na^+^ or Ca^2+^) on the *in vitro* adsorption of ^85^Sr to M-InsP6 (M = Zn or La)

Sr solutions (300 ppm at final concentration) containing 10 kBq/mL of ^85^Sr in 20 mM HEPES buffer (pH 7.4) containing 10 mM Na^+^, 20 mM Na^+^, 50 mM Na^+^, 10 mM Na^+^ + 10 mM Ca^2+^, and 10 mM Na^+^ + 40 mM Ca^2+^ were prepared by dissolution of NaCl or CaCl_2_. M-InsP6 samples (10 mg) were suspended in 1 mL of each Sr solution. After shaking the suspensions at 1,000 rpm and 37°C for 1 h, the binding ratio of each sample to M-InsP6 was determined using the methods described above.

### Biodistribution experiments after oral administration of ^85^Sr with pretreatment of Zn-InsP6

Experiments with all animals (*N* = 59) were conducted in strict accordance with the Guidelines for the Care and Use of Laboratory Animals of Kanazawa University. The animal experimental protocols used were approved by the Committee on Animal Experimentation of Kanazawa University. The animals were housed (4 mice in one polycarbonate cage) under conventional conditions with free access to food and water at 23°C with a 12-hour alternating light/dark schedule. Autoclaved wood chips were applied as bedding material. The behavior of the mice during the experimental period was normal.

Mice were randomly divided into three groups. In a Zn-InsP6 administration group (*N* = 16), Zn-InsP6 suspension in 5% glucose aqueous solution (30 mg / 0.5 mL) was orally administered into 6-week-old male ddY mice (23–30 g, Japan SLC, Inc., Hamamatsu, Japan) using disposable flexible-type animal-feeding-needles (Fuchigami, Muko, Japan).

In an InsP6 administration group (*N* = 18), InsP6 solution in 5% glucose aqueous solution (20 mg / 0.5 mL) was orally administered. In a control group (*N* = 25), 0.5 mL of 5% glucose aqueous solution was orally administered. The number of mice of each group was optimized for ethical reasons, balancing between the number of animals required for statistical reasons for analysis and minimizing the number of mice. Then, just after administration of either Zn-InsP6 suspension, InsP6 solution, or glucose aqueous solution, saline solution of ^85^SrCl_2_ (37 kBq / 100 μL) was orally administered. All animals showed no signs of illness following the administration of the compounds. Mice were sacrificed by decapitation at 1, 4, 24, and 48 h post-administration. Tissues of interest were removed and weighed, and radioactivity counts were determined. From 12 h pre-administration to 24 h post-administration, the fasting was carried out. No animal died prematurely (i.e. besides euthanasia) due to the experimental procedures.

### Radiation dose estimates for ^90^Sr

Radiation dose was calculated from the data of the biodistribution experiments using ^85^Sr as described previously [16]. Namely, for estimation of the radiation dose absorbed by the bone marrow, the percent dose of radioactivity per gram tissue in the bone marrow was assumed to be 30% of the percent dose of radioactivity per gram tissue in the blood. The red marrow mass was assumed to be 25% of blood volume. The blood, bone, and muscle mass of mice were assumed to be 8%, 5%, and 48% of body weight, respectively. According to the International Commission on Radiological Protection (ICRP), an equal distribution of the radionuclide to trabecular and cortical bone was assumed [17]. The non-decay-corrected activity from each source organ was converted to a percentage of the injected radioactivity. The area under each organ’s activity curve from time zero to infinity was calculated by extrapolation of the biodistribution data. To correct for the different ratios of organ to total body weights in mouse and in human, we used the following organ correction factor (CR):

$$CR={\left(organmass/totalbodymass\right)}_{human}/{\left(organmass/totalbodymass\right)}_{mouse}$$

According to the values, the radiation doses for ^90^Sr were calculated for an adult male patient using OLINDA 1.0 software (Vanderbilt University) [18].

### Statistical evaluation

A one-way analysis of variance (ANOVA) followed by Dunnett’s post hoc test compared to the control group was used for experiments of cation effects on the *in vitro* adsorption and biodistribution experiments.

Results were considered statistically significant at *p* < 0.05.

## Results

### Experiments of *in vitro* ^85^Sr adsorption by Zn-InsP6 and La-InsP6 (Langmuir adsorption model)

Adsorption capacity of Zn-InsP6 and La-InsP6 for strontium (Sr) was evaluated by the Langmuir model. The Langmuir equation is expressed as:

$$q=\frac{aq_{max}C_{eq}}{1+aC_{eq}}$$ (1)

where *q* (mg / g) represents Sr binding per Zn-InsP6 and La-InsP6, *C*_eq_ (mg / L) is the equilibrium concentration of Sr, *q*_max_ (mg / g) is the maximum sorption capacity, and *a* (L / mg) is the sorption constant.

**Fig 2:**
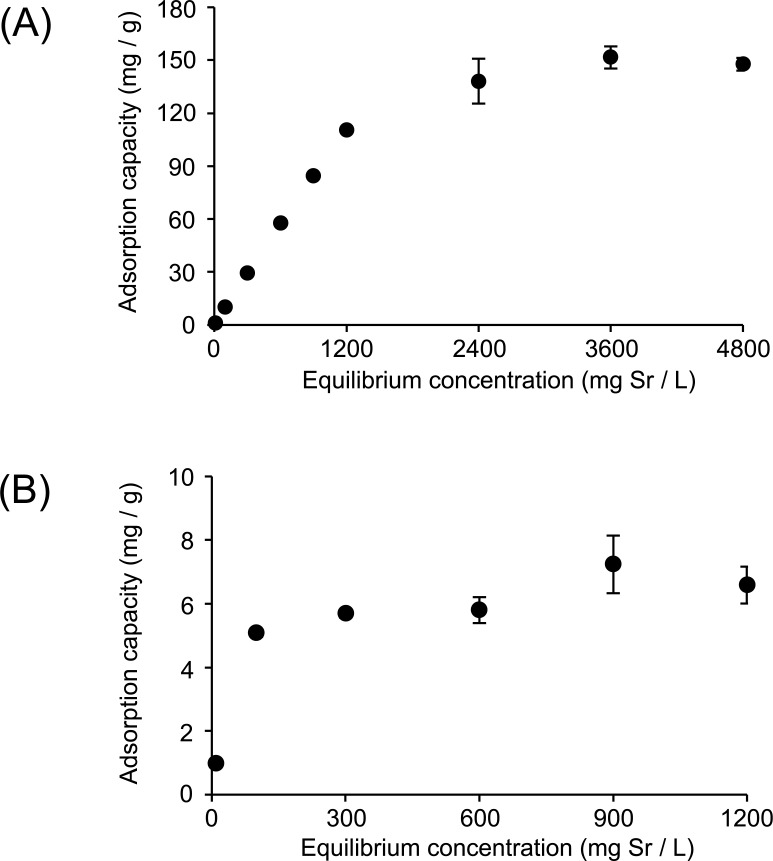
Langmuir model. Adsorption capacity of (A) Zn-InsP6 and (B) La-InsP6 to strontium. Data are expressed as the mean ± SD of three samples.

As shown in Fig 2, the amount of strontium bound to Zn-InsP6 and La-InsP6 increased linearly at low strontium concentrations, and plateaued at high strontium concentrations, indicating saturation of the adsorption sites. These data meet the requirements of the Langmuir adsorption model, indicating the correctness of the assumption that the adsorbate acts as a uniform surface with finite identical binding sites characterized by monolayer adsorption of the adsorbate. The maximum sorption capacity of Zn-InsP6 and La-InsP6 was estimated as 133.7 and 6.4 mg Sr/g, respectively.

### Effects of cations (Na^+^ or Ca^2+^) on *in vitro* adsorption of ^85^Sr^2+^ by Zn-InsP6 and La-InsP6

**Fig 3:**
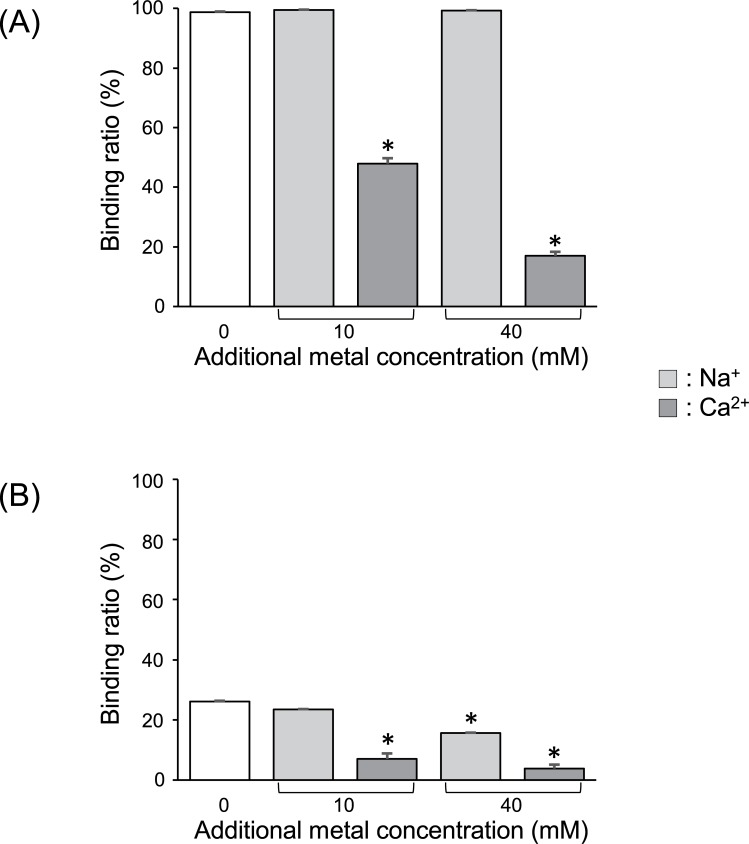
Adsorption of ^85^Sr to IP6 complexes in the presence of cations. Binding ratios of ^85^Sr^2+^ to (A) Zn-InsP6 and (B) La-InsP6 in the presence of Na^+^ or Ca^2+^. Zero additional metal concentration (white columns) means using HEPES buffer containing 10 mM Na^+^. Data are expressed as the mean ± SD of three samples. Significance was determined using one-way ANOVA followed by Dunnett’s post hoc test (*p < 0.05 vs. Control (HEPES buffer containing 10 mM Na^+^)).

Fig 3 shows how other cations affect the binding of strontium to Zn-InsP6 and La-InsP6. The presence of Na^+^ decreased the amount of strontium bound to La-InsP6, but not the amount bound to Zn-InsP6. On the other hand, the presence of Ca^2+^ substantially decreased the amount of strontium bound to both complexes, and this effect depended on the Ca^2+^ concentration.

### Biodistribution experiments

To evaluate whether Zn-InsP6 can enhance the elimination of ^85^Sr from the body *in vivo*, ^85^SrCl_2_ was orally administrated to mice in “treatment groups” just after oral administration of Zn-InsP6 suspension or InsP6 solution. The biodistribution of radioactivity in the mice after 1, 4, 24, and 48 h of oral administration of ^85^SrCl_2_, with or without pretreatment with Zn-InsP6 or InsP6, are reported in Tables 1–4. Pretreatment with Zn-InsP6 significantly decreased the accumulation of radioactivity in the bone and the blood at the early time points after oral administration of ^85^SrCl_2_, relative to the non-treatment control group. These results indicate that Zn-InsP6 inhibited the absorption of ^85^Sr^2+^ from the intestine into the bloodstream and enhanced the excretion of ^85^Sr^2+^ in the feces. At 48 h after administration of ^85^SrCl_2_, the radioactivity levels in all study groups were low in all tissues except bone.

**Table 1:** Biodistribution of radioactivity in mice at 1 h after oral administration of ^85^SrCl_2_ with pretreatment of 5% glucose aqueous solution (control), InsP6 in 5% glucose aqueous solution, or Zn-InsP6 in 5% glucose aqueous solution.

Tissue	Control	InsP6	Zn-InsP6
Blood	0.47 (0.25)	0.05 (0.04)**	0.05 (0.03)**
Liver	0.26 (0.14)	0.05 (0.05)*	0.08 (0.08)*
Kidney	0.48 (0.25)	0.06 (0.04)**	0.07 (0.05)**
Small-intestine^†^	48.5 (13.7)	31.7 (6.08)	49.7 (22.8)
Large-intestine^†^	13.6 (15.3)	0.90 (0.82)	24.7 (19.5)
Spleen	0.18 (0.09)	0.03 (0.03)**	0.02 (0.02)**
Pancreas	0.36 (0.19)	0.03 (0.02)**	0.05 (0.03)**
Lung	0.61 (0.34)	0.13 (0.07)*	0.12 (0.08)*
Heart	0.25 (0.14)	0.02 (0.02)**	0.03 (0.02)**
Stomach^†^	5.44 (6.43)	48.8 (2.67)***	12.1 (5.66)
Bone (Femur)	13.5 (9.61)	0.79 (0.69)*	1.07 (0.39)*
Muscle	0.16 (0.11)	0.01 (0.02)*	0.02 (0.03)*
Brain	0.06 (0.04)	0.00 (0.00)**	0.00 (0.00)*

Data are expressed as % injected dose per gram tissue. Each value represents the mean (±SD) for four (Zn-InsP6 group and InsP6 group) or six (control group) animals.

^†^Data are expressed as % injected dose.

Significance was determined using one-way ANOVA followed by Dunnett’s post hoc test (*p < 0.05, **p < 0.01, ***p < 0.001 vs. Control).

**Table 2:** Biodistribution of radioactivity in mice at 4 h after oral administration of ^85^SrCl_2_ with pretreatment of 5% glucose aqueous solution (control), InsP6 in 5% glucose aqueous solution, or Zn-InsP6 in 5% glucose aqueous solution.

Tissue	Control	InsP6	Zn-InsP6
Blood	0.13 (0.08)	0.13 (0.07)	0.04 (0.01)
Liver	0.17 (0.19)	0.10 (0.04)	0.03 (0.01)
Kidney	0.17 (0.08)	0.15 (0.06)	0.06 (0.02)
Small-intestine^†^	9.43 (9.21)	21.5 (16.8)	2.47 (2.64)
Large-intestine^†^	36.1 (20.6)	37.8 (27.9)	81.5 (12.3)*
Spleen	0.07 (0.07)	0.06 (0.03)	0.03 (0.01)
Pancreas	0.17 (0.17)	0.10 (0.03)	0.04 (0.01)
Lung	0.20 (0.09)	0.58 (0.31)*	0.06 (0.02)
Heart	0.08 (0.07)	0.09 (0.03)	0.00 (0.01)*
Stomach^†^	3.93 (5.00)	17.2 (8.89)*	3.33 (6.60)
Bone (Femur)	15.4 (12.5)	6.00 (2.56)	3.27 (1.32)
Muscle	0.09 (0.06)	0.07 (0.02)	0.01 (0.01)
Brain	0.05 (0.05)	0.03 (0.01)	0.02 (0.00)

Data are expressed as % injected dose per gram tissue. Each value represents the mean (±SD) for four (Zn-InsP6 group and InsP6 group) or six (control group) animals.

^†^Data are expressed as % injected dose.

Significance was determined using one-way ANOVA followed by Dunnett’s post hoc test (*p < 0.05, **p < 0.01, ***p < 0.001 vs. Control).

**Table 3:** Biodistribution of radioactivity in mice at 24 h after oral administration of ^85^SrCl_2_ with pretreatment of 5% glucose aqueous solution (control), InsP6 in 5% glucose aqueous solution, or Zn-InsP6 in 5% glucose aqueous solution.

Tissue	Control	InsP6	Zn-InsP6
Blood	0.06 (0.02)	0.05 (0.01)	0.04 (0.01)
Liver	0.05 (0.02)	0.04 (0.01)	0.03 (0.01)
Kidney	0.09 (0.03)	0.06 (0.01)	0.06 (0.03)
Small-intestine^†^	2.16 (2.21)	1.32 (1.30)	1.32 (1.12)
Large-intestine^†^	12.7 (10.2)	4.76 (2.18)	12.1 (5.97)
Spleen	0.03 (0.03)	0.05 (0.01)	0.04 (0.05)
Pancreas	0.07 (0.02)	0.08 (0.04)	0.04 (0.01)
Lung	0.09 (0.03)	0.07 (0.02)	0.05 (0.01)*
Heart	0.04 (0.02)	0.03 (0.01)	0.03 (0.01)
Stomach^†^	1.74 (2.93)	0.70 (0.80)	0.21 (0.28)
Bone (Femur)	19.4 (9.14)	17.5 (6.47)	10.2 (1.81)
Muscle	0.08 (0.08)	0.06 (0.01)	0.03 (0.03)
Brain	0.04 (0.02)	0.05 (0.03)	0.03 (0.01)

Data are expressed as % injected dose per gram tissue. Each value represents the mean (±SD) for four (Zn-InsP6 group and InsP6 group) or six (control group) animals.

^†^Data are expressed as % injected dose.

Significance was determined using one-way ANOVA followed by Dunnett’s post hoc test (*p < 0.05, **p < 0.01, ***p < 0.001 vs. Control).

**Table 4:** Biodistribution of radioactivity in mice at 48 h after oral administration of ^85^SrCl_2_ with pretreatment of 5% glucose aqueous solution (control), InsP6 in 5% glucose aqueous solution, or Zn-InsP6 in 5% glucose aqueous solution.

Tissue	Control	InsP6	Zn-InsP6
Blood	0.01 (0.01)	0.02 (0.01)	0.01 (0.00)
Liver	0.01 (0.00)	0.01 (0.00)	0.00 (0.00)
Kidney	0.02 (0.03)	0.03 (0.02)	0.02 (0.01)
Small-intestine^†^	0.06 (0.02)	0.07 (0.04)	0.03 (0.01)
Large-intestine^†^	0.11 (0.05)	0.13 (0.07)	0.06 (0.01)
Spleen	0.02 (0.03)	0.02 (0.01)	0.00 (0.00)
Pancreas	0.02 (0.02)	0.02 (0.02)	0.01 (0.01)
Lung	0.02 (0.02)	0.02 (0.02)	0.01 (0.01)
Heart	0.01 (0.01)	0.01 (0.01)	0.02 (0.01)
Stomach^†^	0.01 (0.01)	0.03 (0.02)	0.02 (0.00)
Bone (Femur)	25.2 (8.97)	26.9 (5.37)	9.85 (1.68)**
Muscle	0.04 (0.05)	0.02 (0.03)	0.01 (0.01)
Brain	0.01 (0.00)	0.03 (0.03)	0.01 (0.01)

Data are expressed as % injected dose per gram tissue. Each value represents the mean (±SD) for from four (Zn-InsP6 group), six (InsP6 group), or seven (control group) animals.

^†^Data are expressed as % injected dose.

Significance was determined using one-way ANOVA followed by Dunnett’s post hoc test (*p < 0.05, **p < 0.01, ***p < 0.001 vs. Control).

### Dosimetry

The absorbed radiation dose of ^90^Sr was extrapolated from the data of the ^85^Sr biodistribution experiments, because ^90^Sr should behave identically to ^85^Sr *in vivo*. Table 5 shows the estimated absorbed radiation doses after oral intake of ^90^SrCl_2_ over a one-month period, with and without pretreatment with Zn-InsP6 or InsP6. The Zn-InsP6 treatment decreased the effective dose equivalent of radiation by approximately one half, relative to the non-treatment group. However, the effective dose equivalent was not decreased by treatment with InsP6 (in the absence of the zinc ion).

**Table 5:** Absorbed dose estimates in human after oral intake of ^90^SrCl_2_ with pretreatment of InsP6 or Zn-InsP6, or non-treatment control.

Tissue	Control	InsP6	Zn-InsP6
Osteogenic cells	1.62E+01	1.75E+01	6.56E+00
Red marrow	5.32E+00	5.74E+00	2.91E+00
Liver	7.84E-04	4.49E-04	2.71E-04
Kidneys	2.72E-03	1.73E-03	1.40E-03
Small intestine	1.17E-01	1.57E-01	7.28E-02
Large intestine	7.99E-01	6.00E-01	1.38E+00
Spleen	7.40E-04	7.34E-04	2.96E-04
Pancreas	6.23E-04	5.51E-04	2.87E-04
Lungs	4.59E-03	7.27E-03	2.05E-03
Heart	1.09E-03	7.58E-04	8.54E-04
Stomach	7.95E-02	1.57E-01	5.67E-02
Brain	2.72E-03	2.70E-04	3.35E-04
Muscle	2.91E-02	9.06E-04	5.24E-04
Effective dose equivalent	1.13E+00	1.28E+00	5.46E-01
Effective dose	8.02E-01	9.43E-01	4.36E-01

Expressed as mSv/MBq.

## Discussion

We recently synthesized complexes of La-InsP6 and Zn-InsP6 as radiocesium decorporation agents. We expected that these insoluble complexes can chelate cesium ions in the gastrointestinal tract, inhibiting their absorption into the bloodstream and enhancing the excretion of radioactive Cs^+^ in the feces. According to the experimental results, Zn-InsP6 and La-InsP6 bind Cs^+^ *in vitro* but were ineffective as decorporation agents *in vivo*, because their binding to Cs^+^ was neither strong nor selective enough [15]. In an *in vitro* study, binding of Cs^+^ by La-InsP6 and Zn-InsP6 was inhibited in the presence of Na^+^, K^+^, or Ca^2+^. Among these inhibiting cations, Ca^2+^ most detrimentally affected the binding of La-InsP6 and Zn-InsP6 to Cs^+^. Since InsP6 is known to have a high binding affinity for Ca^2+^ [19], and given the similar chemical properties of Ca^2+^ and Sr^2+^, we assumed that InsP6 can bind a strontium ion more strongly than a cesium ion. Thus, we hypothesized that La-InsP6 and Zn-InsP6 could work as decorporation agents for radioactive strontium.

In the *in vitro* binding experiments, Zn-InsP6 showed higher strontium ion adsorption capacity than La-InsP6 (Fig 2). This difference may derive from the inherent characteristics of the two ions, La^3+^ and Zn^2+^, when forming chelation complexes with InsP6. Whereas Zn^2+^ can form tetracoordinate and hexacoordinate chelation complexes, La^3+^ can only form hexacoordinate complexes. It is speculated that La-InsP6, which should be a strong polymer complex with a dense structure formed by hexacoordination, has limited room for accommodating chelated Sr^2+^. In contrast, Zn-InsP6 has a flexible structure formed by tetracoordination, with more space available for Sr^2+^ binding. These scenarios might explain the higher adsorption capacity of Zn-InsP6 as compared to La-InsP6. Based on the results of the *in vitro* experiments, we performed *in vivo* experiments using Zn-InsP6 alone.

In the Zn-InsP6 treatment group, much lower radioactivity was observed in the blood 1 h after ^85^Sr^2+^ administration when compared to the untreated control group (Table 1). This evidence suggests that Zn-InsP6 prominently inhibited absorption of ^85^Sr^2+^ into the bloodstream from the intestine, due to the adsorption of ^85^Sr^2+^ by the insoluble Zn-InsP6 complex. Since ^85^Sr^2+^ transported by blood preferably accumulates into bone in the whole body, mice in the Zn-InsP6-treated group showed much lower radioactivity in their femurs than mice in the non-treatment control group. Therefore, Zn-InsP6 may be a promising ^90^Sr decorporation agent. Bone accumulation of high-energy beta-particle-emitting radionuclides such as ^90^Sr greatly affect the radiation dose in bone marrow. Moreover, as the biological half-life of strontium in bone tissue is extremely long, strontium accumulated in bone maintains its detrimental effects over a long period [20]. Thus, ^90^Sr bone accumulation is the most important factor determining the effective dose of radiation (Tables 4 and 5).

As InsP6 can bind Sr^2+^, InsP6 alone may also work as a strontium decorporation agent. To test this idea, we performed the biodistribution experiment on an additional control group that had been pre-treated with InsP6. According to the results, InsP6 seemed to inhibit the absorption of ^85^Sr^2+^ into the bloodstream and the accumulation of radioactivity in bone because mice treated with InsP6 displayed lower radioactivity in their blood and bone in the early time points than mice in the non-treatment control group. However, 48 h after administration of ^85^Sr^2+^, the radioactivity biodistributions were almost identical in the InsP6-treated mice and the non-treated mice (Table 4). This evidence indicates that InsP6 alone delayed the movement of ^85^Sr^2+^ along the gastrointestinal tract, but did not prevent its absorption into the bloodstream. In other words, the water-soluble InsP6 (without metal ions) failed as a strontium decorporation agent.

In a previous report, we evaluated *Chlorella*—a genus of single-cell green algae that grow in fresh water—as a decorporation agent for radioactive strontium [21]. In that study, the maximum strontium sorption capacity of *Chlorella* was estimated as 9.06 mg Sr/g. In biodistribution experiments after oral administration of ^85^SrCl_2_ in mice, pretreatment with *Chlorella* significantly decreased the accumulation of radioactivity in bone compared to the control group, but the effects of *Chlorella* showed less than expected ability to inhibit strontium absorption into the bloodstream from the gastrointestinal tract. The efficacies of Zn-InsP6 and *Chlorella* are not easily comparable because the appropriate maximum dose for each compound is not understood. Nevertheless, adsorption of radiostrontium in the intestine and inhibition of its absorption into the bloodstream were anticipated to occur through similar mechanisms by both species. The maximum adsorption capacity of Zn-InsP6 was estimated as 133.7 mg Sr/g, much higher than that of *Chlorella*. This result was reflected in the data of *in vivo* mouse experiments: pretreatment with Zn-InsP6 prominently decreased the accumulation of radioactivity in mouse bones, whereas the inhibitory effects of *Chlorella* were not very pronounced. Since *Chlorella* is a health food, and its safety is highly guaranteed, we cannot simply conclude that Zn-InsP6 is a better option than *Chlorella*. However, Zn-InsP6 is certainly an effective decorporation agent.

In conclusion, these results indicate that the insoluble Zn-InsP6 complex adsorbs radioactive strontium in the gastrointestinal tract, inhibits its absorption into the bloodstream, and enhances its excretion in the feces. Consequently, extrapolating from the results of the biodistribution experiments conducted in mice, the estimated effective dose of ^90^Sr radiation in a human treated with Zn-InsP6 is approximately half of that of the control group. Therefore, Zn-InsP6 presents as a promising ^90^Sr decorporation agent.

## Supporting information

S1 File ARRIVE check list. (DOCX)
