# Supplementary material for: Complexes of myo-inositol-hexakisphosphate (InsP6) with zinc or lanthanum to enhance excretion of radioactive strontium from the body
Source: PLoS One. 2018 Apr 3;13(4):e0195067. doi: 10.1371/journal.pone.0195067 (PMC5882106; doi:10.1371/journal.pone.0195067)

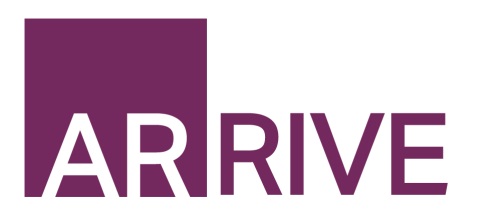


The ARRIVE Guidelines Checklist

Animal Research: Reporting In Vivo Experiments

Carol Kilkenny^1^, William J Browne^2^, Innes C Cuthill^3^, Michael Emerson^4^ and Douglas G Altman^5^

*^1^The National Centre for the Replacement, Refinement and Reduction of Animals in Research, London, UK, ^2^School of Veterinary Science, University of Bristol, Bristol, UK, ^3^School of Biological Sciences, University of Bristol, Bristol, UK, ^4^National Heart and Lung Institute, Imperial College London, UK, ^5^Centre for Statistics in Medicine, University of Oxford, Oxford, UK.*

|  | | ITEM | RECOMMENDATION | Section/ Paragraph |
| --- | --- | --- | --- | --- |
| 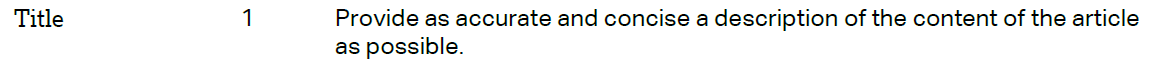 | | | Title |  |
| 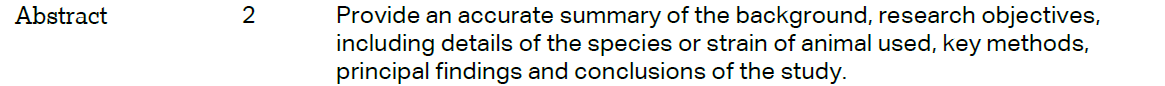 | | | Abstract |  |
| INTRODUCTION | | |  |  |
| 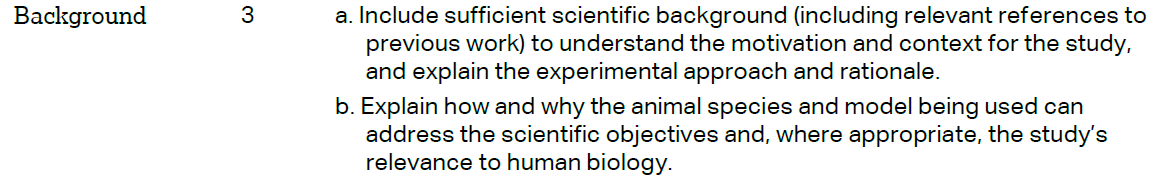 | | | Page 4 Line 2 - Page 5 Line 18  Page 5 Line 18 - Page 6 Line 2 |  |
| 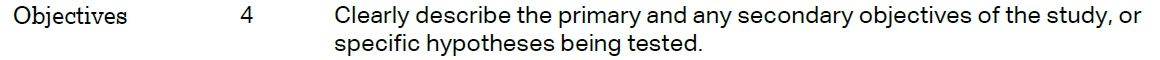 | | | Page 5 Line 8-16 |  |
| METHODS | | |  |  |
| 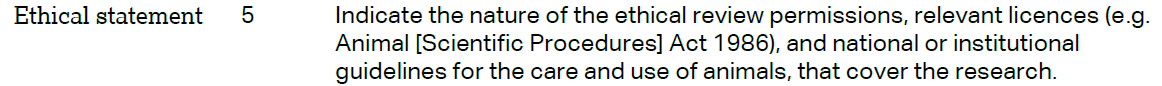 | | | Page 8 Line 9-17 |  |
| 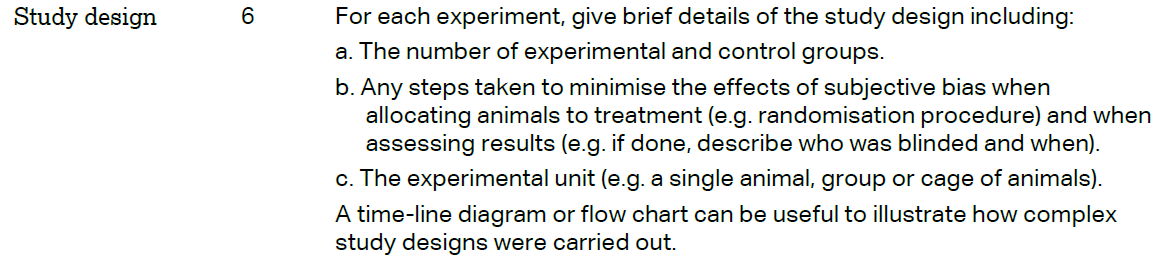 | | | a. Page 8 Line 17 - Page 9 Line 8  b. Page 8 Line 18  c. a single animal |  |
| 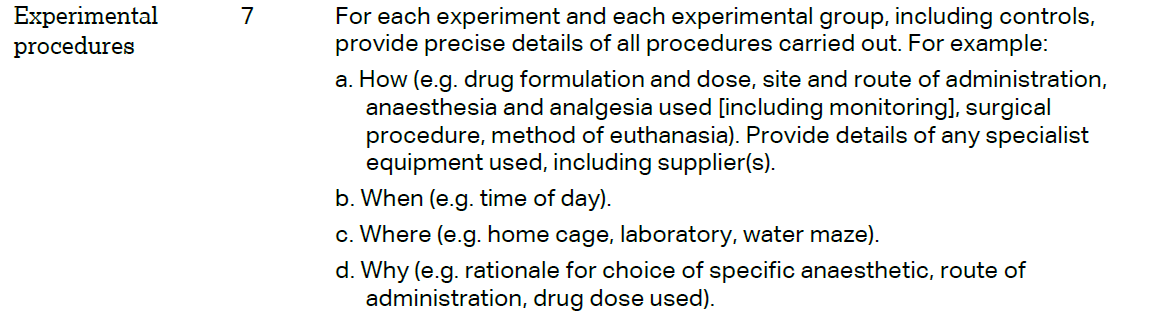 | | | Page 8 Line 18 - Page 9 Line 18 |  |
| 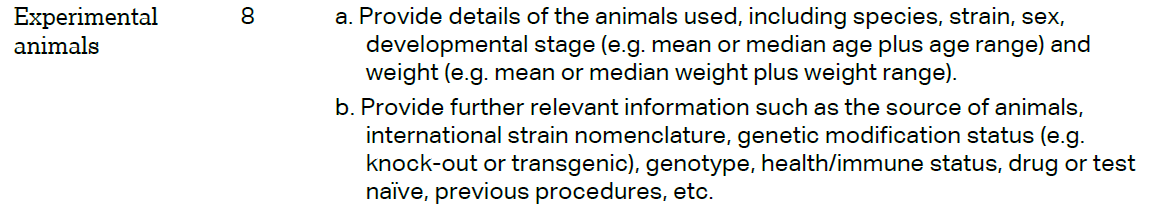 | | | Page 9 Line 2 - 3 |  |

The ARRIVE guidelines. Originally published in *PLoS Biology*, June 2010^1^

| 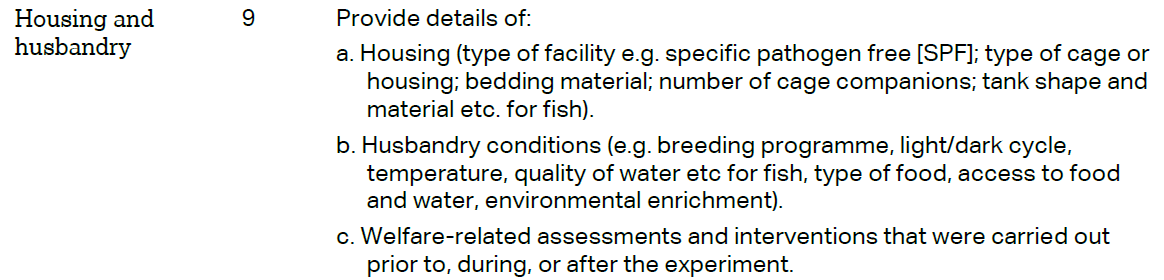 | a. Page 8 Line 13-16  b. Page 8 Line 13-15  c. Not relevant | |
| --- | --- | --- |
| 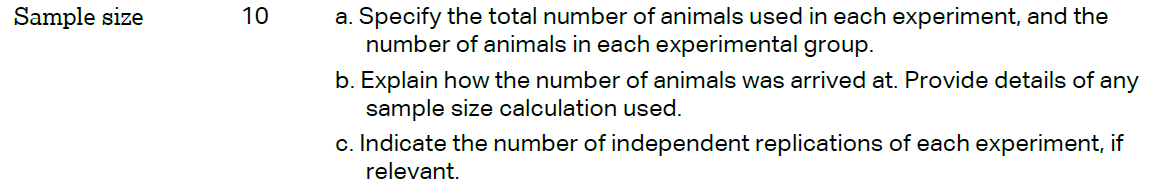 | a. Tables 1-4  b. Page 9 Line 8-10  c. Not relevant | |
| 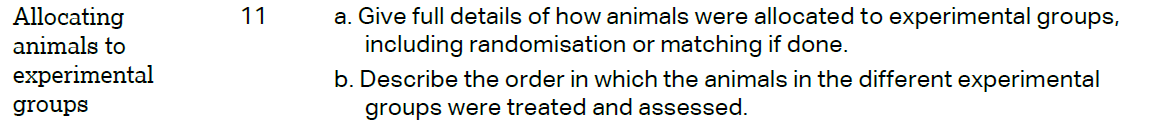 | a. Page 8 Line 18  b. Not relevant | |
| 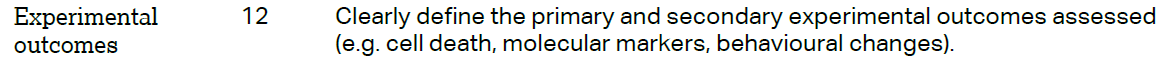 | Page 9 Line 13-14 | |
| 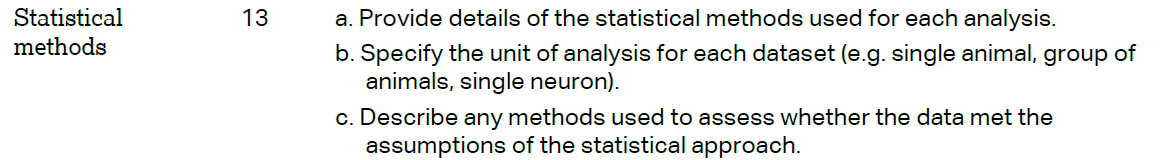 | a. Not relevant  b. Not relevant  c. Not relevant | |
| RESULTS |  | |
| 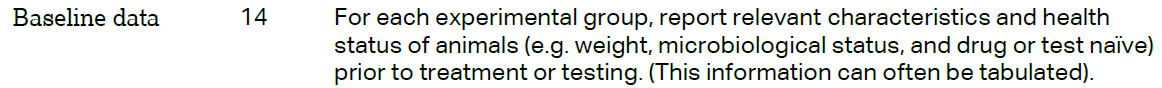 | Page 9 Line 3 | |
| 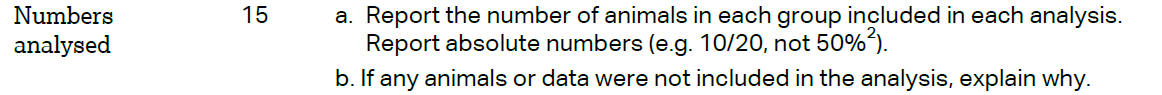 | a. Tables 1-4  b. Not relevant | |
| 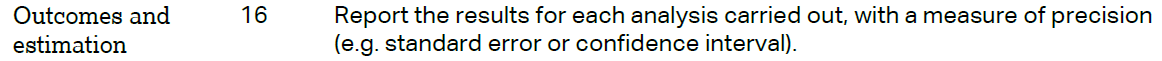 | Tables 1-4 | |
| 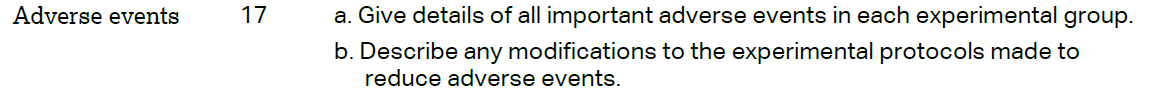 | Not relevant | |
| DISCUSSION |  | |
| 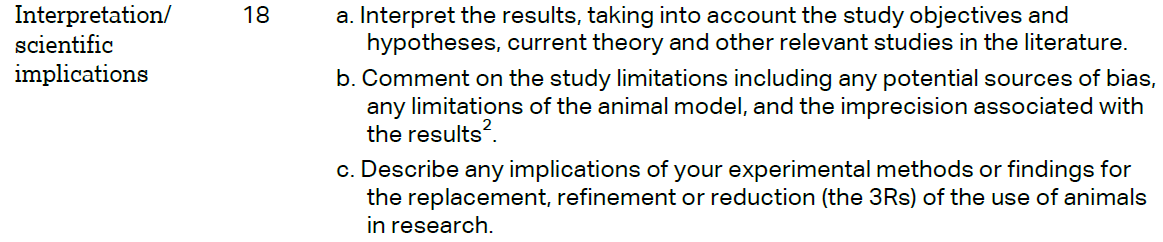 | Page 16 Line 6 - Page 19 Line 15  b. Not relevant  c. Not relevant | |
| 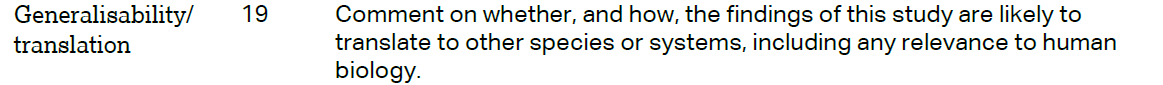 | Page 17 Line 3 - 6 | |
| 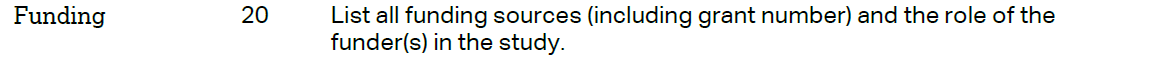 | | Not relevant |


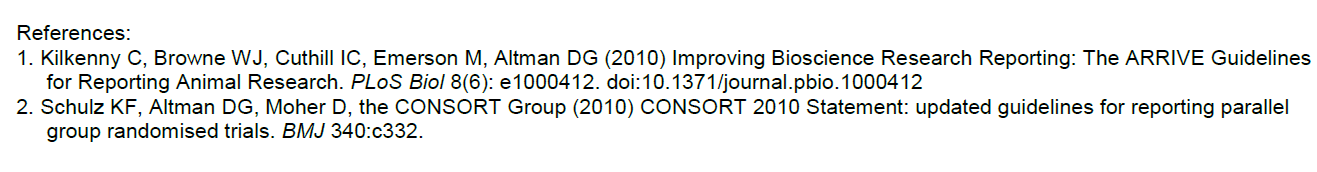

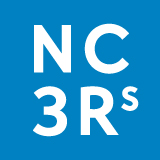

Supplement: S1 File — (DOCX) [file pone.0195067.s001.docx]
